# Prediction of Glycated Hemoglobin Levels at 3 Months after Metabolic Surgery Based on the 7-Day Plasma Metabolic Profile

**DOI:** 10.1371/journal.pone.0109609

**Published:** 2014-11-10

**Authors:** Hyuk Nam Kwon, Yeon Ji Lee, Ju-Hee Kang, Ji-ho Choi, Yong Jin An, Sunmi Kang, Dae Hyun Lee, Young Ju Suh, Yoonseok Heo, Sunghyouk Park

**Affiliations:** 1 College of Pharmacy, Natural Product Research Institute, Seoul National University, Sillim-dong, Gwanak-gu, Seoul, Korea, 151-742; 2 Department of Family Medicine, Obesity center, Inha University Hospital, Inha University School of Medicine, Shinheung-dong, Jung-Gu, Incheon, Korea, 400-712; 3 Department of Pharmacology and Hypoxia-related disease Research, Inha University Hospital, Inha University School of Medicine, Shinheung-dong, Jung-Gu, Incheon, Korea, 400-712; 4 Department of Medicine, Inha University Hospital, Inha University School of Medicine, Shinheung-dong, Jung-Gu, Incheon, Korea, 400-712; 5 Department of Biostatistics, Inha University Hospital, Inha University School of Medicine, Shinheung-dong, Jung-Gu, Incheon, Korea, 400-712; 6 Department of General Surgery, Inha University Hospital, Inha University School of Medicine, Shinheung-dong, Jung-Gu, Incheon, Korea, 400-712; National Research Council of Italy, Italy

## Abstract

Metabolic surgery has been shown to provide better glycemic control for type 2 diabetes than conventional therapies. Still, the outcomes of the surgery are variable, and prognostic markers reflecting the metabolic changes by the surgery are yet to be established. NMR-based plasma metabolomics followed by multivariate regression was used to test the correlation between the metabolomic profile at 7-days after surgery and glycated hemoglobin (HbA1c) levels at 3-months (and up to 12 months with less patients), and to identify the relevant markers. Metabolomic profiles at 7-days could differentiate the patients according to the HbA1c improvement status at 3-months. The HbA1c values were predicted based on the metabolomics profile with partial least square regression, and found to be correlated with the observed values. Metabolite analysis suggested that 3-Hydroxybutyrate (3-HB) and glucose contributes to this prediction, and the [3-HB]/[glucose] exhibited a modest to good correlation with the HbA1c level at 3-months. The prediction of 3-month HbA1c using 7-day metabolomic profile and the suggested new criterion [3-HB]/[glucose] could augment current prognostic modalities and help clinicians decide if drug therapy is necessary.

## Introduction

Diabetes mellitus (DM) is a worldwide disease causing serious health burden with the incidence of approximately 366 million in 2011. [1] A primary risk factor for type 2 diabetes mellitus (T2DM) is obesity and 90% of T2DM patients are either overweight or obese, as defined by body mass index (BMI) of 25 kg/m^2^ or 30 kg/m^2^, respectively. [2] The relative risks of diabetes are 40 times higher when BMI increases above 35 kg/m.^2^ [3] However, obesity seems to be less prevalent in T2DM patients in Asia, [4] suggesting that obesity occurs differently according to population. The main therapeutic modality of T2DM is pharmacotherapy accompanying lifestyle modification, [5] but it is for the control of hyperglycemia, rather than the cure of DM. To date, the only treatment options that have been demonstrated to manage DM without medication are pancreatic transplantation for type 1 DM [6] and surgical treatment for T2DM [7].

Bariatric surgery such as Roux-en Y gastric bypass (RYGB), originally used as a treatment for morbid obesity (>40 kg/m^2^), also showed profound effects on various comorbidities of obesity including DM, [8] hyperlipidemia, hypertension and obstructive sleep apnea. [9] Because bariatric surgery had effects on a variety of metabolic syndrome, it is also called metabolic surgery, [10] and includes several variants: RYGB, duodenojejunal bypass (DJB), [11] and ileal transposition. [12] Metabolic surgery can lead to diabetes resolution, defined by lower levels of glycated hemoglobin (HbA1c) and fasting plasma glucose, occurring within days to weeks after surgery. Although the success rate of DM resolution is variable and long-term follow-up data are limited, a meta-analysis showed that 79.3% of patients exhibited diabetic resolution after RYGB, with 98.9% of patients showing improvement. [13] In addition, recent studies show that the surgical treatments outperform the conventional medical therapies in managing overall diabetic manifestation. [7], [14] Despite these merits, the mechanism of improvement of diabetes by metabolic surgery has been elusive. It has been argued that the improvement of diabetes was due to the reduction in body weight, [15] but diabetes resolution occurred before the improvement in obesity. [8] Studies also demonstrated that metabolic surgery can confer improved glycemic control not only on obese but also on non-obese (<30 kg/m^2^) patients. [16] Therefore, the metabolic surgery seems to improve diabetes in a weight loss-independent way, and the molecular studies related to the detailed mechanism are anticipated.

As DM is essentially a metabolic disease, proper approaches to follow the disease course or the prognosis of treatment options should involve the monitoring of metabolite molecules. In fact, recent applications of metabolomics on DM have discovered useful markers for the alteration of pathophysiologic factors, prognosis prediction or identification of risk factors of the disease. [17], [18] For the metabolic surgeries, metabolomics studies have found that changes in serum aromatic or branched amino acid levels and gut microorganism-derived metabolites may contribute to the improvement of the glycemic control. [19] Still, these marker metabolites are yet to be tested in evaluating the outcomes of the metabolic surgery, and their mechanistic implications are not firmly established. At this point, it is desired to have metabolic markers relevant for the prognostication of the metabolic surgery, as the degree of diabetic improvement can vary significantly. As NMR-based metabolomics has been applied to developing new diagnostic techniques or to predicting the outcomes of drug treatment, [20], [21] we applied it to discovering metabolites related to the different outcomes of metabolic surgery, to understanding the metabolic changes mechanistically related to diabetic improvement, and to providing an approach for the prognosis prediction of glycemic control after metabolic surgery.

## Materials and Methods

### Patients

We recruited 22 patients who were to undergo metabolic surgery for uncontrolled diabetes at the department of general surgery at Inha University Hospital, Incheon, Korea. All agreed with surgical treatment for weight loss or diabetic control, and the expected benefits and risks were explained thoroughly. Written informed consent was obtained from each study participant upon enrollment and prior to the start of the study. The study protocol conforms to the ethical guidelines of the 1975 Declaration of Helsinki, and was approved by the institutional review board at the Inha University Medical School and Hospital (2009-1473) and Seoul National University (1205/001-010). For patient criteria and surgical method, see Materials and Methods S1.

### Sample preparation and NMR measurement

Whole blood was centrifuged at 2,000 g for 10 min at 4°C, and the plasma was collected and immediately stored at −80°C until the NMR experiment. Plasma samples were thawed at room temperature and 250 µL plasma was mixed with 250 µL of NMR buffer based on deuterium oxide (D2O). The final buffer concentration of the plasma sample contained 100 mM potassium phosphate (pH 7.0) and 1 mM 2,2-dimethyl-2-silapentane-5-sulfonate (DSS) as a chemical shift reference. [22] After the buffer mixing, the sample was centrifuged at 20,000 g for 5 min at 4°C, and 480 µL of the sample was transferred to a 5 mm NMR tube. Standard clinical laboratory tests were performed at Inha University hospital. 3-Hydroxybutyrate measurement was done separately using GC-MS after the metabolomics data analysis with the remaining plasma samples kept in a −80°C freezer.

The ^1^H NMR spectra of the plasma samples were measured with a 500 MHz NMR spectrometer (Bruker Biospin, Avance 500) operating at a 500.13 MHz proton NMR frequency at 25°C using Carr-Purell-Meiboom-Gill (CPMG) pulse sequence (cpmgpr1d) with 400 µs of total spin echo delay (Korea Basic Science Institute, Ochang, Korea). The ^1^H spectral FID was collected with 8K complex data points within 30 ppm spectral width during 128 number of scans. The spectra were processed with 0.3 Hz exponential line broadening and zero-filling followed by manual phase and baseline correction. All the spectra were referenced against the DSS signal. The metabolites were identified using Chenomx (Spectral database; Edmonton, Canada) and an in-house built metabolite library. The entire data set of the normalized spectra is given in Fig. S1.

### Multivariate data analysis

All the obtained NMR spectra were Fourier transformed, phase corrected and baseline corrected manually and the resulting spectra were binned at 0.0147 ppm to reduce the signal complexity. After the spectral binning, the data were normalized against total integration values and 1 mmol/L DSS, and then converted into an ASCII text file. The binning, normalization and data conversion were done by an in-house Perl script. For multivariate statistical analysis, the water and EDTA regions were excluded. The converted text file was imported into SIMCA-P version 11.0 (Umetrics, Umeå, Sweden), OriginPro 8 (OriginLab Corporation, Northampton, USA) and R (The R Foundation for Statistical Computing, Vienna, Austria) for statistical analysis. The binned data were mean-centered with Pareto scaling, and orthogonal projections to latent structure-discriminant analysis (OPLS-DA) was performed with one predictive and two orthogonal components, as reported previously. [23] Prediction of improved and non-improved group was performed based on the NMR data obtained 7 days after surgery using both leave-one-out and leave-seven-out analyses. The partial least square (PLS) regression was performed to predict the HbA1c values based on the metabolic profile from the NMR data. The uses of each software are recapitulated as follows: Perl script: numeric data binning and normalization; SIMCA-P 11.0: data scaling, orthogonal projections to latent structure discriminant analysis (OPLS-DA), partial least square (PLS); OriginPro 8, R: regression modeling, correlation analysis and student’s t-test.

## Results

We summarized the basal characteristics and demographics in Table 1, and the changes of clinical parameters after metabolic surgery in Table 2. When we applied the HbA1c value of 7.0 as the cut-off value for the glucose control, [24] about half of the patients (n = 10) showed an improved glycemic control during the follow-up period (3 months), whereas the others (n = 12) did not. Therefore, we categorized the former group as the improved group, and the latter as the non-improved group. The decrease in BMI and AC after the surgery was significant in both groups, with the significance being larger in the non-improved group (*P* of ∼10^−3^ for the non-improved vs. 10^−2^ for the improved). Only the improved group showed normalized oral glucose tolerance test results at 3 months after surgery, even though both group had significant reduction in BMI and AC. Furthermore, one of the patients exhibited a large improvement in HbA1c value with essentially the same BMI at 3 months after surgery with basal BMI (HbA1c from 8.9% to 6% at 3 months; BMI from 23.5 to 23.2). In the other extreme, a patient with large BMI reduction (from 32.4 to 26.7 at 3 months) had a worsened HbA1c value at 3 months after surgery (from 7.7% to 8.7% at 3 months). Therefore, the different prognosis is unlikely dependent on the differential weight loss. Then, we postulated that the improvement might result from the early post-operative changes of individual “metabolic signature”, since diabetes is essentially a metabolic disorder. There are several ways in describing the metabolic status of an individual, but serum metabolomics should be very relevant to this purpose with its comprehensive coverage of metabolites of whole body. Therefore we applied NMR-based metabolomics to these patients' plasma collected at seven days after surgery to see if we can correlate the midterm prognosis at 3 months with this earlier metabolic signature.

**Table 1:** Demographics and pre-operative baseline characteristics (N = 22).

Parameter	Total(N = 22)	Improved*(n = 10)	Non-Improved(n = 12)	P-value^6^
Age (years)	45.2±9.3	39.8±9.2	49.8±6.9	0.009
Sex				0.305#
Male (n (%))	9 (40.9)	3	6	
Female (n (%))	13 (59.1)	7	6	
Diabetes Duration (years)	7.4±4.5	5.1±4.1	9.4±3.9	0.019
Anti-hypertensive	10 (45.5)	6	4	0.206#
medication (n (%))				
BMI^1^ (Kg/m^2^)	27.4±5.3	30.8±5.6	24.6±3.1	0.004
AC^2^ (cm)	89.7±11.6	97.1±12.7	83.5±5.8	0.009
HbA1c^3^ (%)	8.2±1.3	7.9±1.1	8.5±1.5	0.266
FBG^4^ (mg/dl)	185.5±58.6	188.2±69.9	183.5±50.4	0.849
PP2^5^ (mg/dl)	271.1±82.7	234.2±69.0	301.8±83.0	0.054
Operation (n (%))				0.0286#
Roux-en Y Gastric bypass	12 (54.5)	8 (80.0)	4 (33.3)	
Duodenojejunal bypass	10 (45.5)	2 (20.0)	8 (66.7)	

*Patients who had glycated hemoglobin percentage less than 7.0% without glucose-lowering agent at 3 months after metabolic operation.

1 Body mass index; the individual's body mass divided by the square of his or her height.

2 Abdominal circumference.

3 Glycated hemoglobin percentage.

4 Fasting blood glucose level.

5 Blood glucose level in 2 hours after a meal.

6 P-value for unpaired t-test or #chi-square test comparing improved group with non-improved group.

**Table 2:** Change of Anthropometric and Metabolic Parameters in improved and non-improved groups.

	Improved*Mean ± SD			P-value^2^	Non-ImprovedMean ± SD			P-value
	Pre-OP(n = 10)	Post-OP7 days(n = 10)	Post-OP3 months(n = 9)		Pre-OP(n = 12)	Post-OP7 days(n = 12)	Post-OP3 months(n = 10)	
BMI (Kg/m^2^)	30.8±5.6	n.a.^3^	27.2±3.0	0.024	24.6±3.1	n.a.	21.7±2.2	<0.001
AC (cm)	97.1±12.7	n.a.	92.4±12.3	0.018	83.5±5.8	n.a.	79.4±5.5	0.002
HbA1c	7.9±1.1	n.a.	5.86±0.38	0.002	8.5±1.5	n.a.	7.79±0.75	0.157
FBG (mg/dl)	188.2±69.9	110.1±29.2	106.1±12.4		183.5±50.4	137.0±22.8	148.2±27.8	
OGTT30^1^ (mg/dl)	277.4±73.1	171.3±48.0	217.5±29.4		294.3±67.8	208.6±42.6	249.7±46.3	
OGTT60^1^ (mg/dl)	326.9±77.3	228.8±49.8	226.7±49.3		371.5±100.8	254.1±54.8	292.2±59.7	
OGTT90^1^(mg/dl)	336.4±83.2	244.8±47.2	164.6±60.3		424.0±82.6	274.8±60.6	277.1±88.1	
OGTT120^1^ (mg/dl)	313.8±90.9	220.6±53.5	106.1±50.4		423.2±89.9	280.4±56.1	249.4±95.0	

*Patients who had glycated hemoglobin percentage less than 7.0% without glucose-lowering agent in 3 months after metabolic surgery.

1 OGTT indicates oral glucose tolerance test at 30, 60, 90, or 120 min after 75 g glucose intake.

2 paired t-test.

3 Not available.

**Figure 1:**
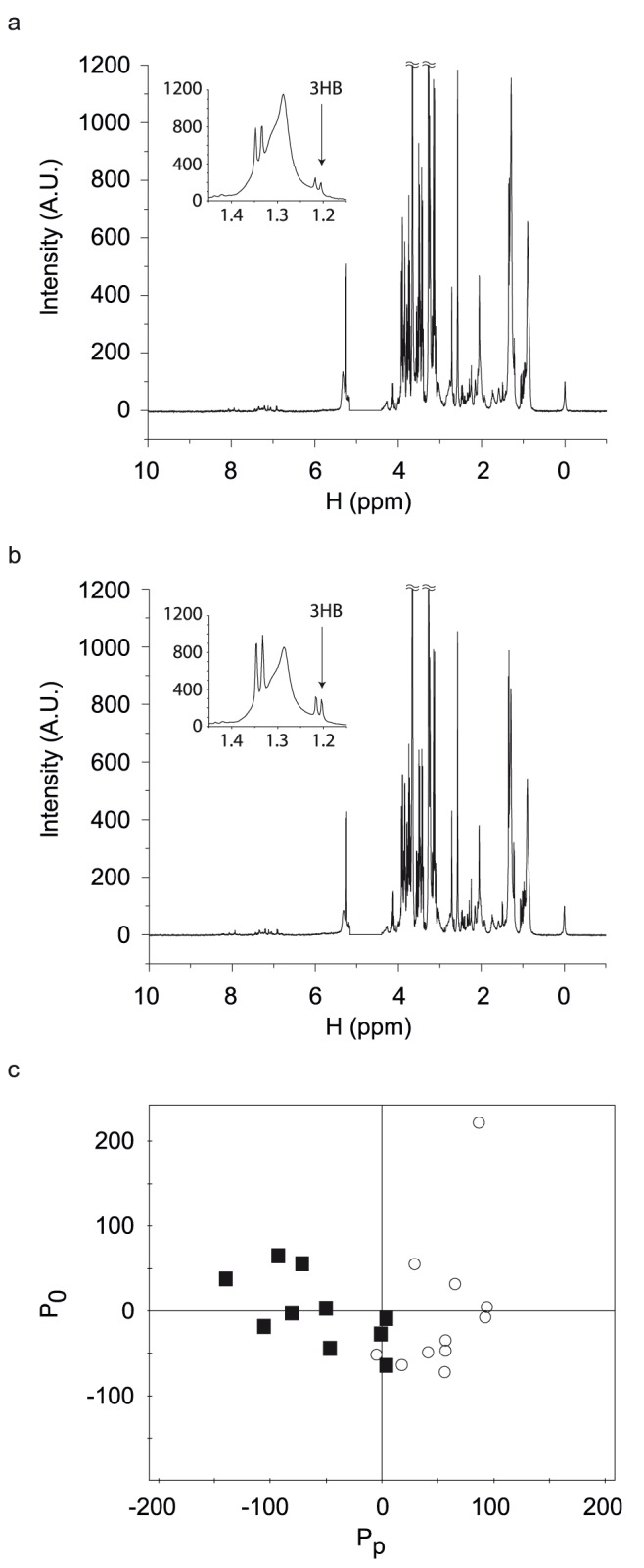
Representative ^1^H NMR spectra of plasma samples and differentiation of improved and non-improved groups. The NMR spectra were taken for plasma samples at 500 MHz. The NMR sample contained final 100 mmol/L potassium phosphate (pH 7.0) and 1 mM DSS as a chemical shift reference. The insets show the 3-HB peak. ^1^H NMR spectra of 7-day post-operative plasma samples from the improved group (a) and from the non-improved group (b). c. OPLS-DA scatter plot was obtained using one predictive and two orthogonal components with R^2^ of 0.671 and Q^2^ of 0.419. Pp and Po represent the predictive component and the orthogonal component respectively. Black filled squares are for the improved group and red filled circles for the non-improved group.

Fig. 1a and b show the NMR data from the two groups of patients which show apparently similar signal patterns. As the NMR data contain many signals coming from a large number of metabolites, we performed multivariate analysis to analyze the data more holistically. We chose OPLS-DA, as it can differentiate patient groups with adjustment of possible confounding variables (i.e. gender, pre-op OGTT and BMI etc.). The differentiation model was obtained with one orthogonal and two predictive components (R^2^ = 0.671 and Q^2^ = 0.419) (Fig. 1c). The analysis shows that the improved and non-improved groups could be separated based on the 7-day post-operative metabolite profiles. Therefore, it seemed that the patient’s metabolic signatures at an earlier post-operative stage (7-days) may be correlated with the post-operative glycemic control in individual patients at 3 months after metabolic surgery.

**Figure 2:**
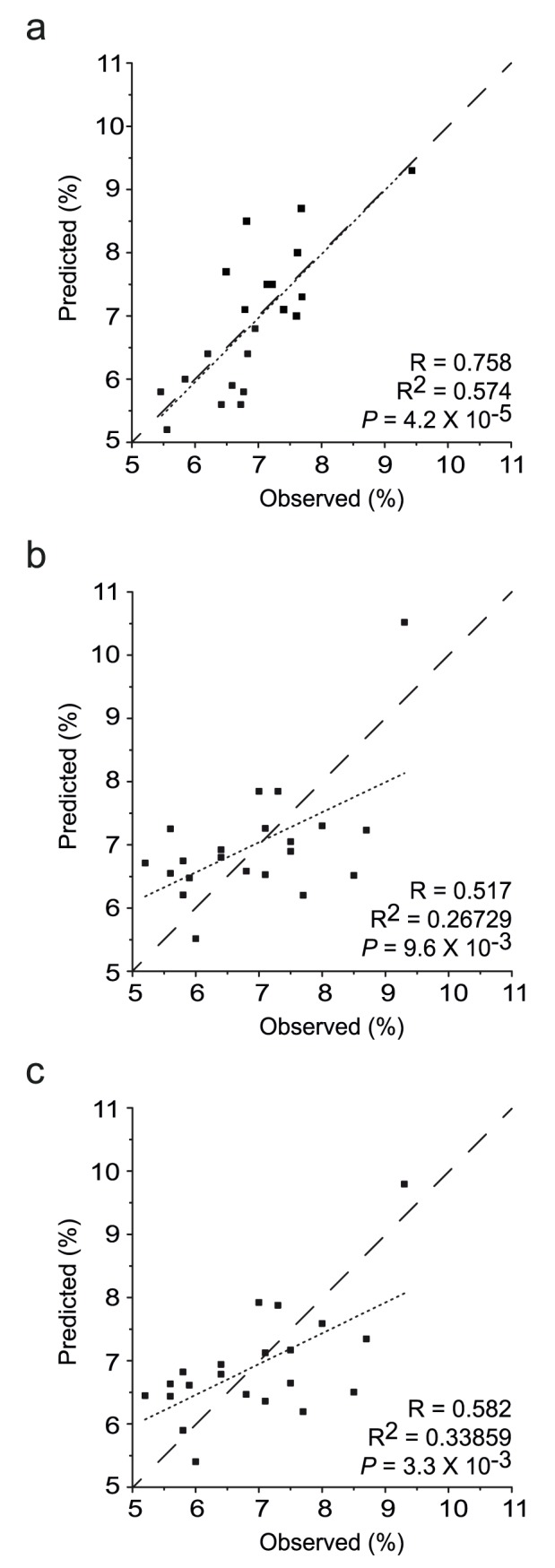
Prediction of HbA1c using PLS multivariate regression. PLS regression models were built with the NMR profile at 7-day time point and the 3-month post-operative HbA1c values. The observed (X-axis) values are actually measured values and the predicted (Y-axis) values are from the PLS regression model obtained with two PLS components. The diagonal dashed line represents the theoretical perfect match, and the dotted line represents the least-square fitted line. Comparison between the observed and predicted values obtained from the training dataset (a), leave-one-out analysis (b), and three-fold cross validation (c). The predicted values in (a) do not represent true prediction since all the data were used in the model building. One (b) or seven (c) samples were left out at a time, and the predictions were made using the model built without the test data to be predicted until every sample was left out once.

To further explore this possibility and to get some quantitative relationship, we performed multivariate regression analysis. We built a PLS regression model using the NMR profile at 7-day time point, and the 3-month post-operative HbA1c values were predicted. Comparison of the predicted and observed HbA1c values, obtained with 2 PLS components, showed reasonably good correlation and predictability (R = 0.758, R^2^ = 0.574, *P* = 4.2×10^−5^) (Fig. 2a). Next, we validated the prediction model by removing one sample at a time and predicting the HbA1c value of the removed sample with a prediction model built without the removed sample. This corresponds to a leave-one-out analysis and can give estimated prediction performance on blind samples. Fig. 2b shows that there is at least a modest correlation between the observed and predicted values for all the samples (R = 0.517, R^2^ = 0.26729, *P* = 9.6×10^−3^). We also performed a 3 fold cross validation (leave-seven-out) which yielded comparable results (R = 0.582, R^2^ = 0.33859, *P* = 3.3×10^−3^) (Fig. 2c). Notably, when we predicted the successful management of the blood glucose based on the predicted HbA1c values (a dichotomic decision: successful management of HbA1c≤7.0; unsuccessful management of HbA1c>7.0), the model correctly classified the management status in 17 out of 21 patients (81% correct) (Fig. 2c).

**Figure 3:**
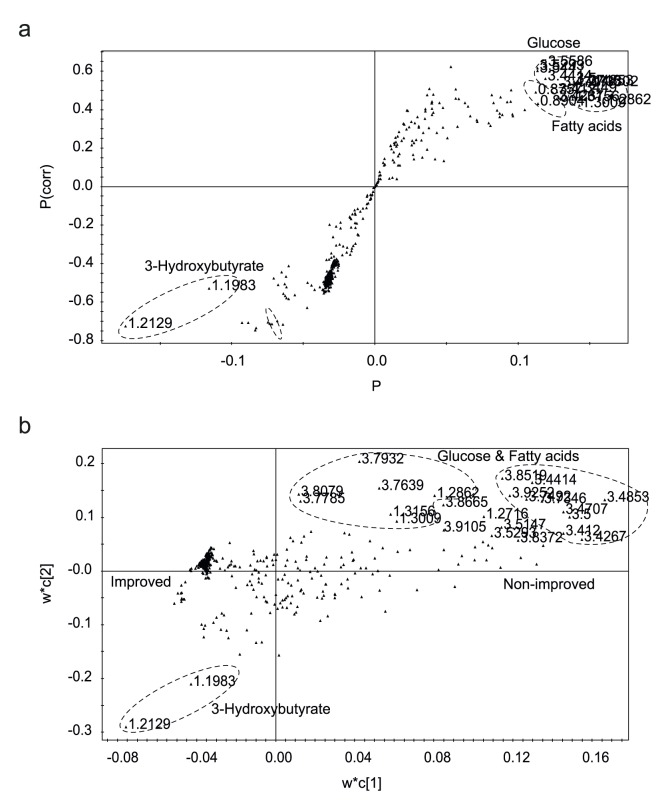
Metabolite marker signals contributing to the differentiation and prediction. a. S-plot analysis showing the correlation and covariation. Metabolites on the upper right corner contribute to the improved group and on the lower left corner contribute to the non-improved group. b. PLS loading plot showing the contribution to the prediction of the HbA1c. Metabolites signals were identified using Chenomx and in-house built metabolite libraries.

**Figure 4:**
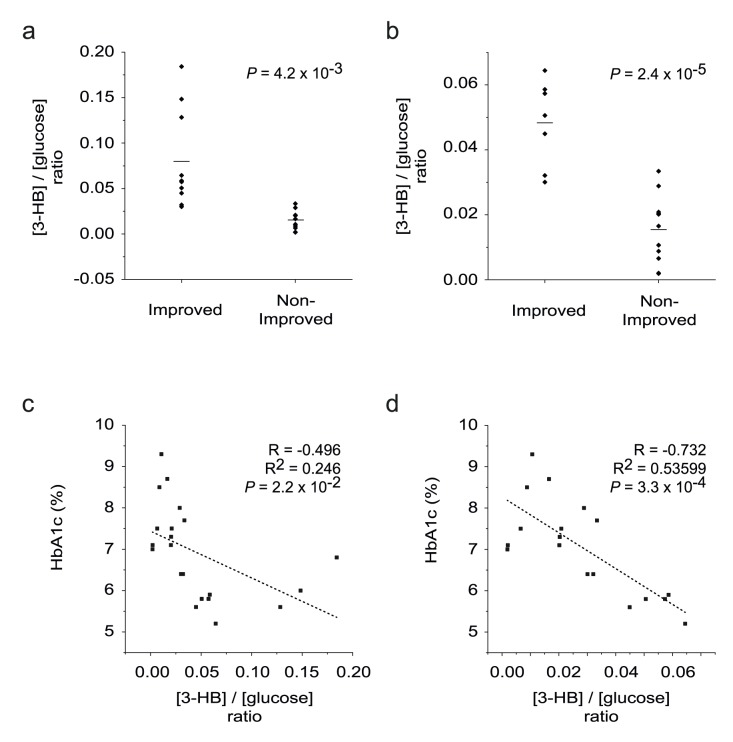
[3-HB]/[glucose] ratio as a criterion for the prognostication of metabolic surgery. 3-HB levels were measured using GC-MS, a standard approach in clinical laboratories. Both 3-HB and glucose levels were from the same plasma samples (7-day post-operative samples) from which the NMR data were obtained. The HbA1c values are from the 3-month post-operative samples. a. The comparison of [3-HB]/[glucose] ratio between the improved and non-improved groups with all the samples. b. The same analysis as (a) after removing the three distinctive outliers with exceptionally high 3-HB values (>1 mM; usual value of ∼0.4 mM). c. Correlation analysis between HbA1c level and [3-HB]/[glucose] ratio with all the samples. d. The same analysis as (c) after removing the three distinctive outliers with exceptionally high 3-HB values (>1 mM; usual value of ∼0.4 mM). The dotted line represents the least-square fitted line ((c) and (d)).

Then, we tried to identify the plasma metabolites at 7-day that contribute most to the difference between successful (improved group) vs. unsuccessful management (non-improved group) of glucose and to the prediction of HbA1c values at 3-month after surgery. To identify the metabolites, we built the S-plot from the OPLS-DA model and loading plot from the PLS-regression model (Fig. 3a and b). Both of these plot show that 3-HB is higher in the improved group, whereas glucose and lipids (LDL, VLDL) are higher in the non-improved group. As 3-HB is a ketone body that can be used as a fuel when glucose is limited, the two molecules are closely related. We tested if the ratio between 3-HB and glucose can be a useful clinical criterion for prognosis prediction of metabolic surgery. For this, we re-measured the 3-HB levels using GC-MS, as it is the standard method used in clinical laboratories. In comparison, NMR is yet to be generally used in clinics. The calculated [3-HB]/[glucose] ratio at 7 days were significantly different between the improved and non-improved groups (*P* = 4.2×10^−3^) (Fig. 4a). The same ratio estimated with NMR data also gave similar trend (Fig. S2). Moreover, the ratio at 7 days showed at least a modest correlation with HbA1c level at 3 months (R = −0.496, R^2^ = 0.246, *P* = 2.2×10^−2^) (Fig. 4c). If we exclude those three distinctive outliers with exceptionally high 3-HB values (>1 mM; usual values of ∼0.4 mM), the difference (*P* = 2.4×10^−5^) (Fig. 4b) and the correlation would become much more robust (R = −0.732, R^2^ = 0.53599, *P* = 3.3×10^−4^) (Fig. 4d). These results suggest that the [3-HB]/[glucose] ratio may be a handy criterion for the prognostication after metabolic surgery, if proven in a larger study.

Encouraged by these results at 3 months, we applied the same procedure to the prediction of a longer term blood glucose control at 12 months post-operative time points. Unfortunately, the number of patients that were followed for 12 months was relatively small (n = 13), therefore, the statistical significance of PLS regression model is lower than that of 3 months. Nevertheless, we believe it is worth providing the results for future reference. Consistent with 3-month results, the PLS model showed a good correlation between predicted and observed HbA1c value at 12 months post-operative time (R = 0.846, R^2^ = 0.71515, *P* = 1.65×10^−4^) (Fig. S3a), and the leave-one-out prediction test gave R = 0.572, R^2^ = 0.32675, and *P* = 2.4×10^−2^ (Fig. S3b).

## Discussion

Growing evidence has suggested that bariatric surgery is able to promote remission of T2DM, and to treat the morbid obesity as well. However, the glycemic control months after surgery within and across studies seems variable, and the indicators for the early decision of the improvement are not currently available. Here, we showed that the metabolic profiles in subjects with improved glycemic control could be differentiated from those without improvement as early as 7 days after metabolic surgery, and that the metabolic profile is correlated with HbA1c values at 3 months after surgery. Initial baseline (pre-operative) and post-operative results suggested that some general clinical parameters such as age, BMI, AC, and diabetes duration are also significantly different between the improved and non-improved groups (Table 1). Therefore, we performed correlation analysis with these possible confounding baseline characteristics against HbA1c level at 3 months. The resulting R values were 0.485 (R^2^ = 0.2348), −0.623 (R^2^ = 0.3883), 0.594 (R^2^ = 0.353), and −0.548 (R^2^ = 0.3003) for duration of diabetes, AC, age, and BMI, respectively (Fig. S4). The analysis also showed that the patients with less age, higher BMI, higher AC and lower diabetic duration are likely to have better prognosis. Compared to these, the correlation analysis using the metabolomic data to predict HbA1c level performed much better than these parameters (R = 0.758, R^2^ = 0.574; Fig. 2a). We believe our data warrant larger studies with more patients for longer follow-up period. If proven in these future studies, the blood metabolomic signature could be used to predict the long term diabetic prognosis with data obtained shortly after the metabolic surgery. As the metabolomic data represent the “individual metabolic signature” of a patient, the approach may be ultimately applied to personalized diabetic management after metabolic surgery based on individual plasma molecular metabolic profiles.

Several studies provided evidence that improvement of T2DM is likely independent of the reduction of body weight by metabolic surgery. [25] Consistent with these, the significance of the post-operative BMI changes in the improved group is weaker than that in the non-improved group (Table 2). In addition, the mean ratio between the BMI change over 3 months by surgery and pre-operative basal BMI in the improved group (0.1169) was actually similar to the non-improved group (0.1179). To test the effects of BMI change in a more quantitative manner, we performed correlation analysis between the BMI changes and HbA1c at pre-operative, post-operative stages and their difference (Fig. S5). The R values from these analyses are all minimal (<0.2), indicating that post-op BMI changes may not have direct implication in diabetic improvement at least up to 3 months. These results are significant, since one of the key questions in this field is the involvement of BMI reduction in the diabetic improvement by metabolic surgery.

Another main finding of our study is that the most relevant metabolic features contributing to the correlation between the 7-day post-operative metabolic profile and 3-month HbA1c value are 3-HB and glucose concentrations. The patients with higher 3-HB and lower glucose concentrations at 7 days after surgery showed better glycemic control later (3 months). While lower glucose concentration may be easily accepted for its correlation with lower HbA1c value, 3-HB may deserve more discussion. 3-HB is a ketone body formed from acetyl CoA during fasting conditions for use in brain or other tissues as fuel. High levels of 3-HB can be present in diabetic ketoacidosis found usually in Type 1 DM or stress conditions after surgery. [26] It should be stressed that the increase in 3-HB in the improved group in our study is not simply due to the surgical stress or fasting, as the increase is relative to the non-improved group and both groups underwent surgery. Significant increases in 3-HB after bariatric surgery as compared to the pre-operative values have been also reported, but the 3-HB level was not implicated in the difference in the diabetic improvement months after the surgery. [25] As 3-HB is a marker of fatty acid oxidation, it is possible that the abrupt change of energy metabolism in T2DM patients by metabolic surgery may acutely reverse the insulin-resistant status. In fact, 3-HB is formed primarily when the energy source for the peripheral organs are shifted to lipid from glucose and, therefore, the early stage metabolic shifts by metabolic surgery may be a key factor for the success of the longer term post-operative glucose control. More mechanistically relevant for the current study are the reports on the direct effects of 3-HB on the insulin sensitivity shown in animal models. [27], [28] Using partial pancreatectomized (Px) rats, a good model for non-obese T2DM, [29] Park et. al., showed that intraperitonial injection of 3-HB into the diabetic rats leads to decreased hepatic glucose output compared to the diabetic controls using hyperinsulinemic euglycemic clamp setting. [27] This improved glucose hometostasis by 3-HB was suggested to be due to the enhanced hepatic insulin signaling, as evidenced by the increased phosphorylation of IRS2 and AKT. In another study, [28] it was shown that intracranial injection of 3-HB into Px diabetic rats also leads to decreased hepatic glucose production and enhanced glucose tolerance through increased hepatic insulin signaling and decreased phosphoenolpyruvate carboxykinase (PEPCK) level, the rate-limiting enzyme for gluconeogenesis. These improved glycemic control by 3-HB was attributed to enhanced leptin and insulin signaling in the hypothalamus rather than increased insulin secretion. Therefore, higher levels of 3-HB at the early days after the bariatric surgery in the improved group in our study might have induced similar enhancement in insulin signaling leading to better glycemic control. With a recent report suggesting that early high levels of 3-HB after metabolic surgery return to normal levels after several months, [30] it seems that 3-HB values before, not after, significant weight loss might be mechanistically related to the diabetic improvement. With this mechanistic relevance and our data showing the correlation with the prognosis at 3 months, the [3-HB]/[glucose] ratio might be a useful prognostic marker for future glycemic control, if our results are reproduced in larger studies. These markers could help clinicians with deciding if early drug therapies are necessary shortly after metabolic surgery for individual patients, ultimately contributing to personalized treatment.

As with any other clinical studies, several limitations of our study should be noted. First, the number of subjects is relatively small, particularly for the subjects followed for 12 months. Therefore, our results could be stepping stones for future larger studies for confirming the clinical applicability. Second, the follow-up period (up to 12 months) was not enough to conclude the long-term (> years) effects of metabolic surgery on glycemic control in T2DM patients. Third, we measured the post-operative, not pre-operative, metabolic profiles to observe the correlation between metabolomics data and diabetic control. Therefore, to determine the baseline characteristics of metabolites affecting the post-operative prognosis, further study will be needed. Finally, we applied different surgical procedures according to clinical decision. However, the focus of this study was not the effects of different surgical procedure, but the discovery of metabolites related to the post-operative glycemic control. Therefore, future studies individually designed for different surgical procedures will be necessary to see the effects of the surgical procedure. Still, it should be noted that our prognosis prediction was obtained irrespective of the surgical procedures, and turned out to be significant.

In conclusion, the NMR-based metabolomics data obtained at 7 days after metabolic surgery could be correlated with the post-operative diabetic improvement according to the HbA1c level at post-op 3-months. The metabolomics data analysis showed that the higher level of [3-HB]/[glucose] ratio at 7 days after surgery can differentiate the patients with or without glycemic control at 3 months after surgery. These results provide evidence for the potential value of NMR-based metabolomics data to assess the heterogeneous results of metabolic surgery in T2DM, and suggest that 3-HB level may be related to the mechanism of the diabetic improvement effect of metabolic surgery. Our study warrants further studies with larger number of subjects followed for the longer term to confirm the mechanisms and/or clinical utility of the biomarkers.

## Supporting Information

Figure S1 **The entire normalized data set is presented as stacked spectra.** The black data set represents the improved group and the red data set represents the non-improved group.
(EPS)

Figure S2 **[3-HB]/[glucose] ratios of the improved and non-improved groups based on NMR spectral integration.** The error bars represent the standard deviation.
(EPS)

Figure S3 **Prediction of HbA1c using PLS multivariate regression: 12-month data.** PLS regression models were built with the NMR profile at 7-day time point and the 12-month post-operative HbA1c values. The observed (X-axis) values are actually measured values and the predicted (Y-axis) values are from the PLS regression model obtained with two PLS components. The diagonal dashed line represents the theoretical perfect match, and the red dotted line represents the least-square fitted line (b). Comparison between the observed and predicted values obtained from the training dataset (a), leave-one-out analysis (b). The predicted values in (a) do not represent true prediction since all the data were used in the model building. One (b) sample was left out at a time, and the predictions were made using the model built without the test data to be predicted until every sample was left out once.
(EPS)

Figure S4 **Correlation analysis between 3-month post-operative HbA1c values and possible confounding baseline characteristics.** The dotted line was obtained with linear least square method. The correlation with HbA1c and duration of diabetes(a), AC(b), Age(c), and BMI(d) are presented.
(EPS)

Figure S5 **Correlation.** Correlation analysis between BMI changes and HbA1c at various times. HbA1c initial is for the pre-operative value (a), HbA1c 3-month for the 3-month value (b), and dHbA1c for the difference between the two (c). The dotted line was obtained with linear least square method. ΔBMI is for the BMI changes at the 3-month post-operative time point as compared to the pre-operative values.
(EPS)

Materials and Methods S1 **Patients criteria and Surgical procedures.**
(DOCX)
