# Supplementary material for: Prediction of Glycated Hemoglobin Levels at 3 Months after Metabolic Surgery Based on the 7-Day Plasma Metabolic Profile
Source: PLoS One. 2014 Nov 10;9(11):e109609. doi: 10.1371/journal.pone.0109609 (PMC4226477; doi:10.1371/journal.pone.0109609)
Supplement: Materials and Methods S1 — Patients criteria and Surgical procedures. (DOCX) [file pone.0109609.s006.docx]

Supporting Information Materials and Methods

<Patients criteria>

Eligible patients met the following criteria: age between 20 and 60 years with uncontrolled type 2 diabetes, and insulin therapy less than 10 years, if taken. We excluded the patients with type 1 diabetes, diabetes due to other endocrine diseases, positive anti-GAD antibody, fasting C-peptide level less than 1.0 ng/mL, and other medical conditions or histories such as pregnancy, severe cardiovascular disease, malignancy, major organ dysfunction, and incongruity for surgery. 22 patients with T2DM were recruited, and fasting blood samples were collected before surgery at indicated time points after metabolic surgery (7 days, 3 months, and 12 months). Patient samples were separated into the “improved” and the “non-improved” groups according to the HbA1c value of 7%. This value is recommended as a reasonable glycemic goal for non-pregnant adults by the American Diabetes Association.[[1](#_ENREF_1)] We did not match the patients based on age or gender for the improved and non-improved groups, since these groups were assigned after the follow-up.

<Surgical procedures>

Patients with BMI over 28 underwent laparoscopic RYGB (n = 12) and those with BMI less than 25 underwent DJB (n = 10). For RYGB, 5 ports and one 3 mm incision for Nathane retractor were employed, and 80 cm of roux and biliopancreatic limb was made. Gastric pouch was formed by 4 or 5 linear stapling, and jejuno-jejunostomy and gastrojejunostomy was made by linear stapler. Leakage test for gastrojejunostomy was performed in all cases. For DJB, all the patients chose the open method although it was left to each patient to choose open or laparoscopic surgery. A median incision of approximately 8 cm was made in upper abdomen. The greater momentum was opened and dissection between the posterior wall of duodenal bulb and head of the pancreas was performed to distal part of duodenal bulb as much as possible. After having the posterior wall of duodenum dissected sufficiently, duodenum was stapled and divided at a distance of at least 4 cm from pylorus. Preservation of the right gastric and right gastroepiploic vessels was successful in all patients. After transecting jejunum 80 cm from the Treitz ligament, end-to-end anastomosis of distal jejunum and duodenum was made. About 80 cm from this anastomosis, end-to-side jejuno-jejunostomy was made. Therefore the common limb began in the lower 160 cm from Treitz ligament.

**Reference:**

1. (2013) Executive summary: Standards of medical care in diabetes--2013. Diabetes Care 36 Suppl 1: S4-10.
